# Patient-Reported Outcomes After Swallowing (SWOARs)-Sparing IMRT in Head and Neck Cancers: Primary Results from a Prospective Study Endorsed by the Head and Neck Study Group (HNSG) of the Italian Association of Radiotherapy and Clinical Oncology (AIRO)

**DOI:** 10.1007/s00455-022-10434-4

**Published:** 2022-05-19

**Authors:** Stefano Ursino, Elisa Calistri, Francesca De Felice, Pierluigi Bonomo, Isacco Desideri, Pierfrancesco Franco, Francesca Arcadipane, Caterina Colosimo, Rosario Mazzola, Marta Maddalo, Alessandra Gonnelli, Giulia Malfatti, Riccardo Morganti, Daniela Musio, Fabiola Paiar

**Affiliations:** 1 Radiation Oncology Unit, University Hospital Santa Chiara, Via Roma 67, 56100 Pisa, Italy; 2 Radiation Oncology Unit, University Hospital La Sapienza, Viale del Policlinico 155, 00161 Rome, Italy; 3 Radiation Oncology Unit, University Hospital Careggi, Largo Brambilla 3, 50134 Florence, Italy; 4 Department of Translational Medicine, University of Eastern Piedmont, Novara, Italy; 5 Department of Oncology, Radiation Oncology, University of Turin, Via Camillo Benso di Cavour 31, 10123 Turin, Italy; 6 Radiation Oncology Unit, S.Luca Hospital, Via Guglielmo Lippi Francesconi 556, 55100 Lucca, Italy; 7 Advanced Radiation Oncology Department, IRCCS Sacro Cuore-Don Calabria Hospital Cancer Care Center, Via Sempreboni 5, 37024 Verona, Italy; 8 Radiation Oncology Unit, Department of Medical and Surgical Specialties, Radiological Science and Public Health, ASST Spedali Civili of Brescia, University of Brescia, Via del Medolo 2, 25123 Brescia, Italy; 9 Department of Clinical and Experimental Medicine, Section of Statistics, Via Roma 67, 56100 Pisa, Italy

**Keywords:** Deglutition, Patient-reported outcomes, MDADI, Head and neck cancer, Cancer-related dysphagia, Treatment-related dysphagia

## Abstract

**Objectives:**

To prospectively investigate changes in M.D. Anderson Dysphagia Inventory (MDADI) scores in patients affected by naso- and oropharynx cancer after definitive radiochemotherapy (ChemoRT) using swallowing organs at risk (SWOARs)-sparing IMRT.

**Methods:**

MDADI questionnaires were collected at baseline and at 6 and 12 months after treatment. MDADI scores were categorized as follows: ≥ 80 “optimal,” 80–60 “adequate,” < 60 “poor” deglutition-related quality of life (QoL) group, and dichotomized as “optimal” vs “adequate/poor” for the analysis. A mean MDADI composite (MDADI-C) change of 10 points was considered as minimal clinically important difference (MCID).

**Results:**

Sixty-three patients were enrolled of which 47 were considered for the analysis. At baseline, 26 (55%) were “optimal” and 21 (45%) were “adequate/poor.” The mean baseline MDADI-C score was 93.6 dropping to 81 at 6 months (*p* = 0.013) and slightly rising to 85.5 at 12 months (*p* = 0.321) for the “optimal” group. Indeed, the mean baseline MDADI-C score was 64.3 rising to 77.5 at 6 months (*p* = 0.006) and stabilizing at 76 at 12 months (*p* = 0.999) for the “adequate/poor” group. A statistically significant but not clinically relevant worsening of the MDADI-C score was reported for the “optimal” group, whereas both a statistically significant and clinically meaningful improvement of the MDADI-C score were reported for the “adequate/poor” group from before to post-treatment.

**Conclusion:**

Our results suggest a doubly clinical benefit of dose optimization to SWOARs to minimize the RT sequalae in patients with a baseline “optimal” deglutition-related QoL and to recover from cancer dysphagia in those with a baseline “adequate/poor” deglutition-related QoL.

**Supplementary Information:**

The online version contains supplementary material available at 10.1007/s00455-022-10434-4.

## Introduction

Head and neck squamous cell carcinoma (HNSCC) is the sixth most common malignancy accounting for approximately 6% of all cancers and for an estimated 1–2% of all cancer-related deaths [1]. Radiotherapy (RT), alone or combined with chemotherapy (ChemoRT), is the mainstay non-surgical treatment despite often being hampered by a non-negligible rate of radiation-induced dysphagia (RID) [2]. A recent study [3] showed RID as having the highest priority among a cluster of quality of life (QoL) RT-related symptoms in a heterogeneous HNSCC population, as also reported by several previous studies [4–6]. For most people, the possibility to enjoy eating helps to achieve a good QoL, whereas labored deglutition, prolonged eating times, and the limited range of foods that can be swallowed can lead to disruption of relationships and social isolation [7].

The scientific community recognizes that clinicians underestimate the consequences of RID and now encourages the use of patient-reported outcomes (PROs) questionnaires [8]. In this regard, the M.D. Anderson Dysphagia Inventory (MDADI) is the most widely recognized PRO metric to assess deglutition-related quality of life (QoL), properly aimed at investigating patients’ perception of deglutition disorders and its related QoL [9].

To date, several cross-sectional studies have reported MDADI outcomes in a heterogeneous HNSCC survivor population but only few studies have investigated the longitudinal changes over time and they have sometimes not included a pre-treatment evaluation [10–15]. Indeed, the latter is of primary importance to detect possible pre-treatment cancer-related dysphagia and to properly interpret post-treatment RT-related dysphagia. This often represents the sum of both factors, combined with the patient’s ability to compensate spontaneously or with targeted therapy [8].

On the other hand, the advancement of intensity-modulated radiotherapy (IMRT) technologies has shown promising results in terms of better deglutition outcomes [16]. The normal swallowing is a very complex process simultaneously involving multiple muscles, such as the base of tongue for the posterior propulsion of the bolus through the pharynx, the pharyngeal constrictors for the cranio-caudal peristalsis of the bolus, and the cricopharyngeal muscle whose opening promotes the passage of the bolus into the upper digestive tract. Also, the suprahyoid musculature contraction for the upward and forward motion of the hyolarynx complex, together with the glottic and supraglottic closure represents the main mechanism to protect the lower airways from possible inhalation. Therefore, if the reduction of radiation dose to parotids and several swallowing organs at risk (SWOARs-sparing IMRT) by generating highly conformal and steep gradient dose distributions around these organs might decrease the percentage of radiation-related deglutition disorders is yet to be elucidated [17].

In this regard, there is a small amount of published data showing a significant reduction of severe deglutition disorders when comparing SWOARs-sparing with standard parotid-sparing IMRT, although outcomes focused on diet texture and feeding tube placement alone [18]. This study aims to report longitudinal patient-reported swallowing outcomes for SWOARs-sparing Chemo-IMRT as part of a multicenter study.

## Materials and Methods

### Study Protocol

The study was approved by the Ethics Committee of the Coordinating Center (Protocol number 42380) and that of each participating center; all patients enrolled in the study accepted the protocol and signed a study-specific informed consent form. The protocol is registered in ClinicalTrials.gov (ID. NCT03448341).

Briefly, patients underwent evaluation by means of MDADI questionnaire at baseline and at 6 and 12 months after treatment.

Briefly, the eligibility criteria included all patients affected with nasopharynx or oropharynx cancers (Stage III–IVA according to 7th TNM Stage Edition), with histologically proven diagnosis of undifferentiated nasopharyngeal-type carcinoma or squamous cell carcinoma, Eastern Cooperative Oncology Group Performance Status (ECOG PS) 0–2, and age < 80 years. Exclusion criteria were as follows: a different tumor site from naso or oropharynx, a different histology from undifferentiated nasopharyngeal type or squamous cell carcinoma, ECOG Performance Status ≥ 3, Stage IVB and C, prior induction chemotherapy or HN treatment (surgery and/or RT), and diagnosis of comorbid conditions potentially compromising basic deglutition function (e.g., demyelinating or degenerative diseases; connective tissue diseases; history of excessive use of benzodiazepines or similar, gastroesophageal reflux resistant at medical therapy and age > 80 years). Patients who underwent salvage surgery at the primary tumor site and those who experienced primary recurrence or metastatic disease within the timeframe of the study were dropped out of the study. Differently, patients who underwent salvage surgery for nodal tumor persistence or recurrence after treatment were not excluded.

### Radiotherapy

Details regarding RT treatment planning criteria are reported in the *Electronic Supplementary Material File*. Briefly, all patients required bilateral neck irradiation and underwent tridimensional image-guided RT (IGRT) and IMRT. The high- and low-risk clinical target volumes (HR-CTVs and LR-CTVs) were defined together with an optional intermediate risk (IR-CTVs), according to HNSG-AIRO guidelines [19]. Corresponding Planning Target Volumes (PTVs) were automatically created by uniform expansions of 0.3 cm. The prescribed radiation doses were 66 Gy, 60 Gy, and 54 Gy given over 30 daily fractions to HR-PTVs, IR-PTVs, and LR-PTVs, respectively. According to the computed tomography (CT)-based delineation guidelines of the swallowing organs at risk (SWOARs) by Christianen et al. [20], 8 different SWOARs (superior, medium, and inferior constrictor muscle; base of tongue; supraglottic and glottic larynx; cricopharyngeal muscle; cervical esophagus) were defined to optimize the IMRT plan for a dose reduction to these structures (SWOARs-sparing IMRT). One radiation oncologist for each participating center was identified for the delineation of SWOARs, assisted by a dedicated radiologist if necessary. The IMRT plans set target prescription goals and spinal cord and brainstem maximum dose (*D*_max_) as the highest priority, whereas SWOARs constraints were set as secondary.

Chemotherapy was given weekly using Cisplatin 40 mg/m^2^ for a maximum of 6 cycles or 100 mg/m^2^ every 3 weeks during the 6-week RT course for a maximum of 3 cycles.

### Evaluation of Dysphagia

All the enrolled patients filled in the MDADI questionnaire at baseline and at 6 and 12 months after ChemoRT. Briefly, the MDADI consists of a self-reported questionnaire with 20 questions and 4 subscales about deglutition-related QoL: Global assessment, single-item MDADI-G; Functional, 5-item MDADI-F; Physical, 8-item MDADI-P; and Emotional, 6-item MDADI-E. Each question includes a 5-point response scale. A composite score (MDADI-C) based on 19 items (excluding the G-item) was applied to evaluate deglutition-specific QoL. All subscale MDADI scores (F, P, and E) and C scores were normalized to values ranging from 20 (extremely low functioning) to 100 (high functioning), with higher scores representing better QoL [9]. In addition, the validated Italian version of MDADI was used [21]. A MDADI score of at least 80 represented an “optimal” deglutition-related QoL, a score between 60 and 80 represented an “adequate” deglutition-related QoL, and a score less than 60 represented a “poor” deglutition-related QoL; this classification based on the MDADI score was used for the intent of our analysis as a post hoc stratification.

A 10-point difference in the MDADI composite score was considered a minimal clinically important difference (MCID) in deglutition-related QoL [22]. Only the patients who filled in all the time interval questionnaires (baseline, 6, and 12 months) were considered for the analysis.

### Statistical Analysis

Categorical data were described by absolute and relative frequencies and continuous data were described by mean and standard deviation. To compare the MDADI score among the three temporal measures, ANOVA for repeated measures stratified for MDADI value at baseline (< 80; ≥ 80) was used and multiple comparisons were performed by the Bonferroni method. Significance was fixed at 0.05. All analyses were carried out by SPSS v.27 technology.

## Results

### Patients

**Fig. 1:**
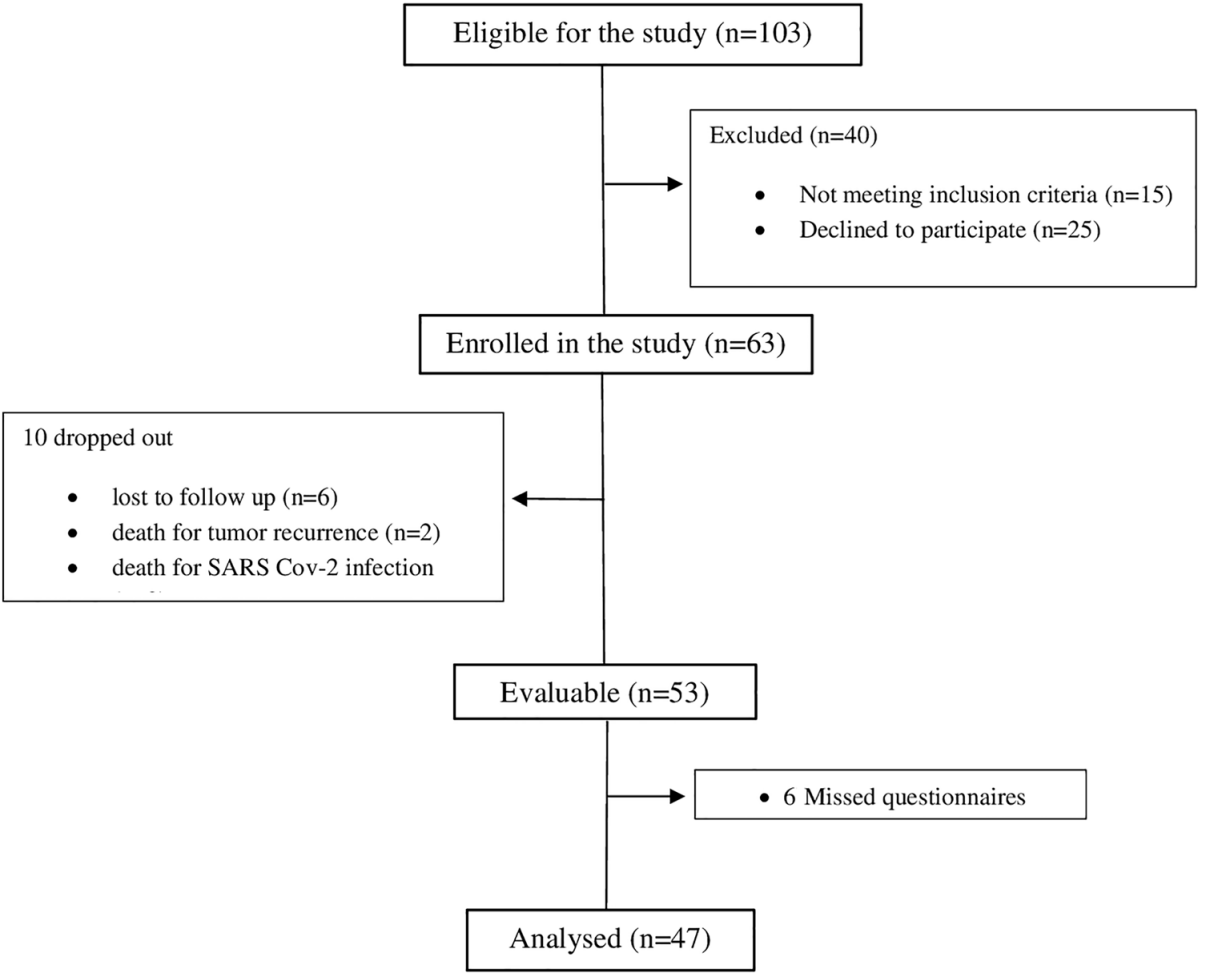
The CONSORT diagram of the study

Between August 2015 and May 2020, among a total of 103 eligible patients, 63 (61.2%) were enrolled from 6 participating centers. Among them, 10 dropped out of the study of which 6 discontinued control visits, 2 died due to tumor recurrence, and 2 died due to SARS Cov-2 infection within 5 months from the end of treatment. Of the 53 remaining evaluable patients, 6 missed to fill in all the questionnaires. Thus, a total of 47 patients were definitively considered for the present final analysis as we decided to consider only those who filled in all the time interval (baseline, 6, and 12 months) questionnaires (Fig. 1). Patients and tumor characteristics are shown in Table 1.

**Table 1:** Patients and tumor characteristics

Characteristics	Statistics
Age, year mean (range)	63 (45–82)
Gender, *n* (%)	
Male	38 (81)
Female	9 (19)
Smoking status	
No	23 (49)
Yes	24 (51)
Alcohol	
No	27 (57)
Yes	20 (43)
Site	
Nasopharynx	5 (11)
Oropharynx	42 (89)
Histology	
SCC	39 (83)
WHO2	2 (4)
WHO3	6 (13)
p16	
neg	8 (17)
pos	28 (60)
Unknown	11 (23)
T stage	
T1	3 (6)
T2	22 (47)
T3	7 (15)
T4	15 (32)
N stage	
N0	4 (8.5)
N1	10 (21.5)
N2	33 (70)
Stage	
III	19 (40)
IVA	28 (60)

Average and range doses to the SPCM, MPCM, IPCM, BOT, SL, GL, CPM, and CE were 58.2 Gy (59.9–32.8 Gy), 55.45 Gy (70.1–31.5 Gy), 44 Gy (62.9–20.3 Gy), 58.6 Gy (70.5-35 Gy), 49.2 Gy (67.1–30 Gy), 39.6 Gy (68–9.6 Gy), 38.1 Gy (54.2–16.7 Gy), and 29 Gy (49.2–11.2 Gy).

Among the 42 oropharyngeal patients, 23 (55%) had tumor localized within the base of tongue, 16 (38%) within the tonsil, 2 (5%) within the soft palate, and 1 (2%) within the posterior pharyngeal wall. Also, 28/42 (67%) were N2 of which 2 (7%) were N2a, 19 (68%) were N2b, and 7 (25%) were N2c. A total of 23/47 (49%) patients underwent triweekly Cisplatin, whereas 24/47 (51%) underwent weekly Cisplatin. Overall, 33/47 (70%) patients completed the planned cycles of CT, whereas 14/47 (30%) did not. Then, 2/47 (4%) patients required feeding tube positioning before RT which was removed at 3 and 9 months after the end of treatment, respectively.

Among the 47 analyzed patients, 5 (10.5%) died of whom 3 (6%) due to tumor recurrence and 2 (4%) to non-cancer-related causes. Overall, 6/47 (13%) patients experienced regional recurrence of which only 1 (2%) died due to nodal recurrence (not amenable for salvage neck dissection), whereas the others underwent salvage neck surgery. Among these patients, only 1 developed concurrent lung metastases and was addressed to systemic treatment.

### MDADI Score Changes

Among the 47 evaluable patients, 26 (55%) were classified as “optimal,” 14 (30%) as “adequate,” and 7 (15%) as “poor” according to the MDADI-C-score at baseline. 25 (53%) were classified as “optimal,” 17 (36%) as “adequate,” and 5 (11%) as “poor” for MDADI-C score at 6 months. Finally, 26 (55.3%) were classified as “optimal,” 18 (38.3%) as “adequate,” and 3 (6.4%) as “poor” for MDADI-C score at 12 months. The mean value and standard deviations (SD) of the MDADI-C-score for the entire patient population were 80.52 (SD 17.65) at baseline, 79.50 (SD 13.00) at 6 months, and 81.20 (SD 13.65) at 12 months, respectively (Table 2).

**Table 2:** MDADI composite score variations for the overall patient population

	Mean	Sd	*p*-value
MDADI-C baseline	80.5	17.7	0.674
MDADI-C 6 months	79.5	13.1	
MDADI-C 12 months	81.2	13.6	

*MDADI* M.D. Anderson Dysphagia Inventory; *C* composite; *sd* standard deviation

**Fig. 2:**
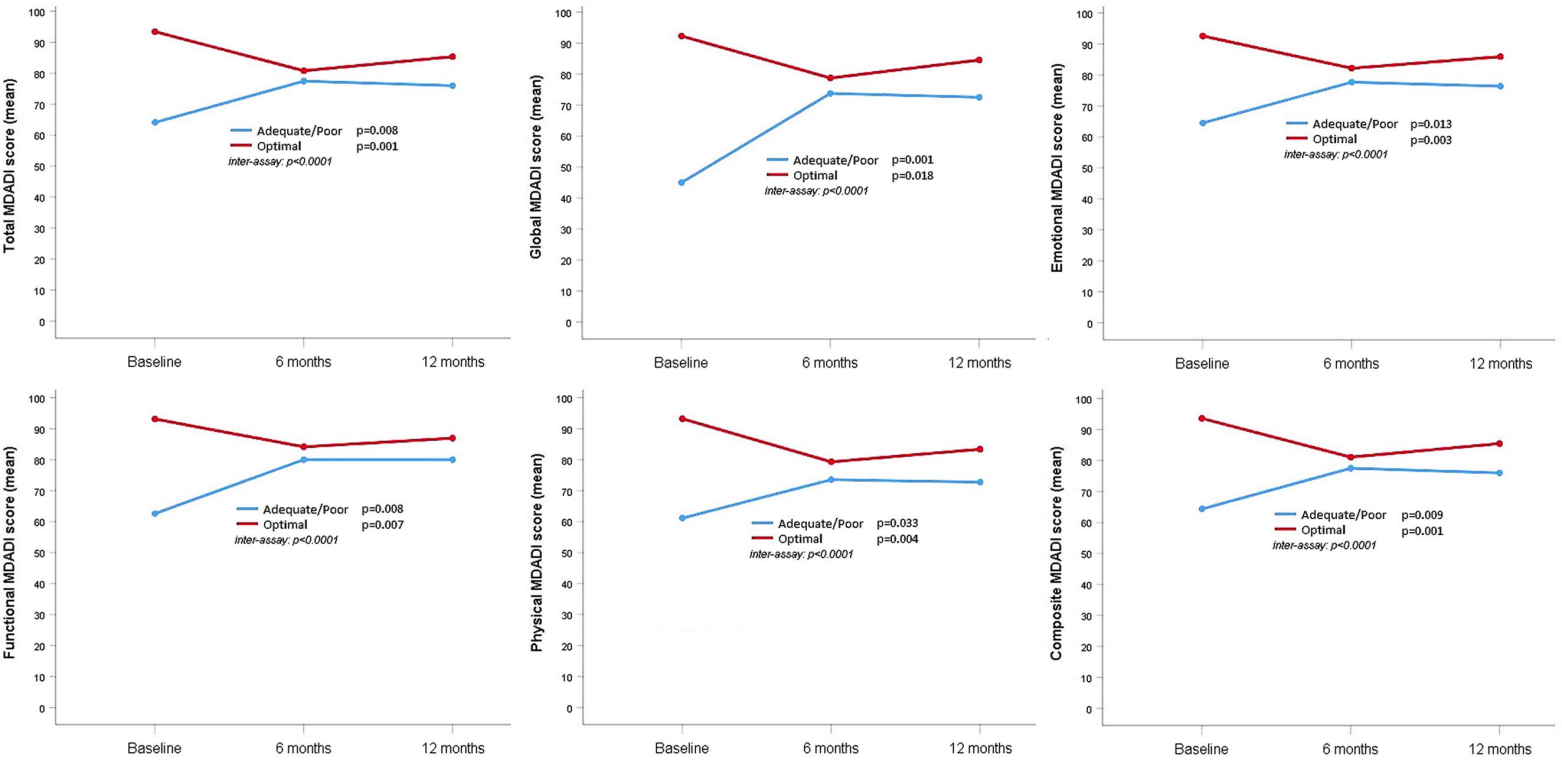
M.D. Anderson Dysphagia Inventory (MDADI) scores trend for the “optimal” and “adequate/poor” group

An overall statistically significant worsening of MDADI-C (*p* = 0.001) and all subscale scores (*p* < 0.05) were reported for the “optimal” group as well as a statistically significant improvement of MDADI-C (*p* = 0.009) and all subscale scores (*p* < 0.05) were reported for the “adequate/poor” group from before to after treatment. The overall MDADI-C and all subscale scores trajectory for both groups of patients are shown in Fig. 2.

In the “optimal” group, mean baseline MDADI-C scores were 93.60, dropping to 81.00 at 6 months (*p* = 0.001) and slightly rising to 85.50 at 12 months (*p* > 0.05) after treatment. On the contrary, in the “adequate/poor” group, the mean baseline MDADI-C score was 64.30 rising to 77.50 at 6 months (*p* = 0.006) and stabilizing at 76.00 (*p* > 0.05) after 12 months. An analogous trend was reported for all subscale scores for both the “optimal” and the “adequate/poor” groups. The MDADI-C score remained statistically significant (*p* = 0.013) but not clinically worsened (93.6 vs 85.5) in the “optimal” group comparing baseline scores to those recorded after ChemoRT. Indeed, the MDADI-C score was statistically significant (*p* = 0.033) and clinically improved (64.3 vs 76.0) in the “adequate/poor” group from before to after ChemoRT. Table 3 shows all other multiple comparisons between scores at different time points.

**Table 3:** Comparison of MDADI scores at different time points

MDADI score	Time	MDADI group	*N*	Mean	SD	*p*-value	Multiple comparisons
Total	(0) baseline	< 80	21	64.10	12.80	0.008	(0) vs (1): *p* = 0.006
	(1) 6 months		21	77.43	11.25		(0) vs (2): *p* = 0.030
	(2) 12 months		21	75.95	12.92		(1) vs (2): *p* = 0.999
	(0) baseline	≥ 80	26	93.38	6.38	0.001	(0) vs (1): *p* = 0.001
	(1) 6 months		26	80.77	14.96		(0) vs (2): *p* = 0.013
	(2) 12 months		26	85.31	13.48		(1) vs (2): *p* = 0.143
Global	(0) baseline	< 80	16	45.00	11.55	0.001	(0) vs (1): *p* = 0.003
	(1) 6 months		16	73.75	26.04		(0) vs (2): *p* = 0.001
	(2) 12 months		16	72.50	22.95		(1) vs (2): *p* = 0.999
	(0) baseline	≥ 80	31	92.26	9.90	0.018	(0) vs (1): *p* = 0.013
	(1) 6 months		31	78.71	21.87		(0) vs (2): *p* = 0.189
	(2) 12 months		31	84.52	21.73		(1) vs (2): *p* = 0.321
**Emotional**	(0) baseline	< 80	20	64.50	12.44	0.013	(0) vs (1): *p* = 0.009
	(1) 6 months		20	77.65	11.86		(0) vs (2): *p* = 0.028
	(2) 12 months		20	76.35	12.42		(1) vs (2): *p* = 0.999
	(0) baseline	≥ 80	27	92.56	7.39	0.003	(0) vs (1): *p* = 0.002
	(1) 6 months		27	82.15	14.60		(0) vs (2): *p* = 0.027
	(2) 12 months		27	85.89	13.95		(1) vs (2): *p* = 0.430
**Functional**	(0) baseline	< 80	14	62.57	12.51	0.008	(0) vs (1): *p* = 0.008
	(1) 6 months		14	80.00	11.53		(0) vs (2): *p* = 0.009
	(2) 12 months		14	80.00	12.84		(1) vs (2): *p* = 0.999
	(0) baseline	≥ 80	33	93.09	8.20	0.007	(0) vs (1): *p* = 0.008
	(1) 6 months		33	84.12	15.12		(0) vs (2): *p* = 0.017
	(2) 12 months		33	86.91	12.74		(1) vs (2): *p* = 0.642
**Physical**	(0) baseline	< 80	22	61.14	13.84	0.033	(0) vs (1): *p* = 0.034
	(1) 6 months		22	73.52	14.87		(0) vs (2): *p* = 0.071
	(2) 12 months		22	72.73	16.46		(1) vs (2): *p* = 0.999
	(0) baseline	≥ 80	25	93.18	7.94	0.004	(0) vs (1): *p* = 0.003
	(1) 6 months		25	79.28	16.24		(0) vs (2): *p* = 0.033
	(2) 12 months		25	83.36	16.55		(1) vs (2): *p* = 0.589
**Composite**	(0) baseline	< 80	21	64.30	12.95	0.009	(0) vs (1): *p* = 0.006
	(1) 6 months		21	77.50	10.84		(0) vs (2): *p* = 0.033
	(2) 12 months		21	76.00	12.57		(1) vs (2): *p* = 0.999
	(0) baseline	≥ 80	26	93.60	6.51	0.001	(0) vs (1): *p* = 0.001
	(1) 6 months		26	81.00	14.67		(0) vs (2): *p* = 0.013
	(2) 12 months		26	85.50	13.22		(1) vs (2): *p* = 0.999

*MDADI* M.D. Anderson Dysphagia Inventory; *N* number; *sd* standard deviation;

### MDADI-C Group Shifts

**Fig. 3:**
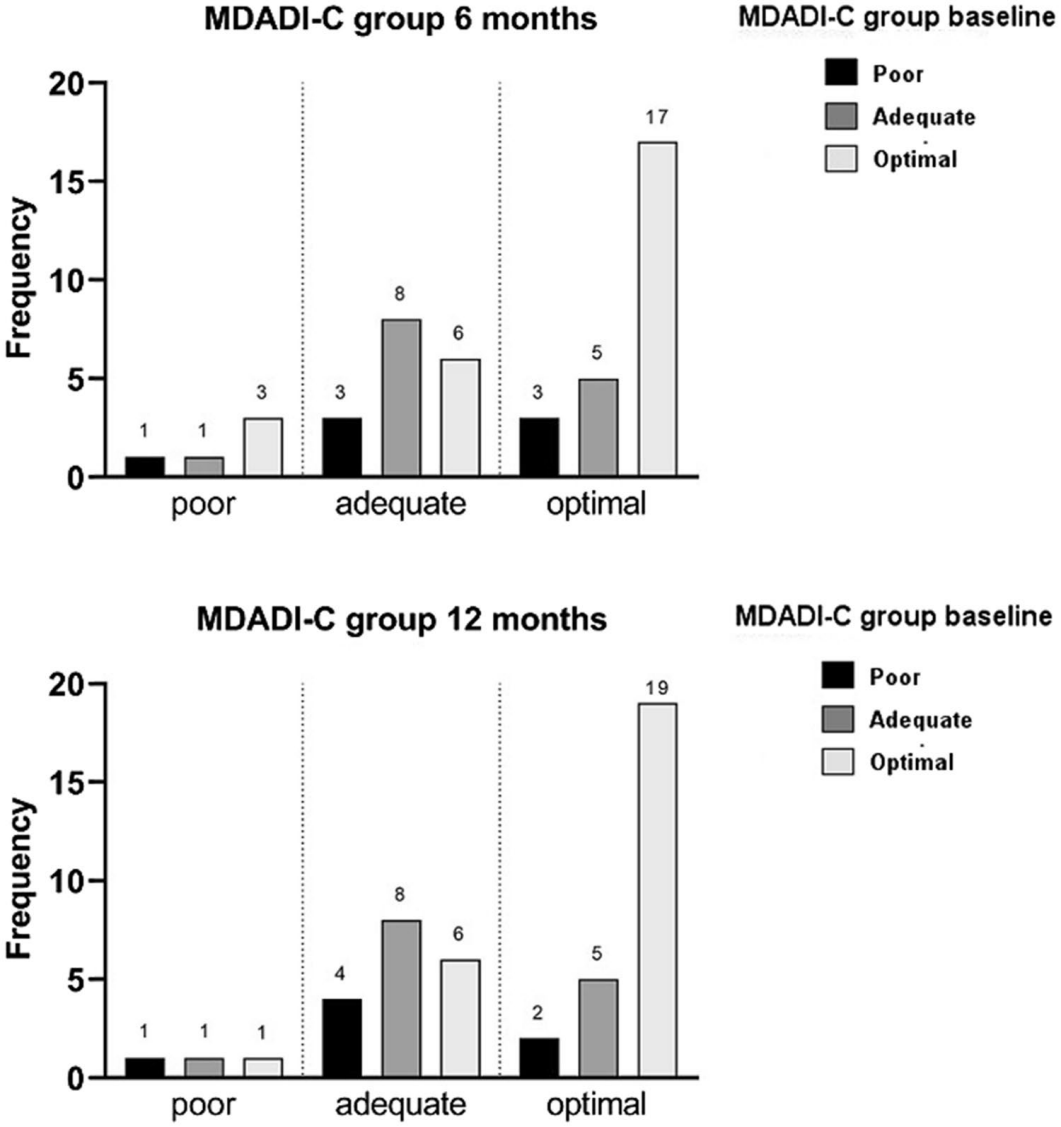
M.D. Anderson Dysphagia Inventory composite (MDADI-C) group shifts for the “optimal,” “adequate,” and poor” group at 6 and 12 months

The trend of the MDADI-C group shifts for the “optimal,” “adequate,” and “poor” groups of patients at different times is shown in Fig. 3.

Specifically, in the “optimal” group, 9**/**26 patients (35%) experienced a downshift of the C-score from baseline to 6 months. Of these, 6 (67%) decreased to “adequate” and 3 (33%) to “poor,” respectively. Again, in the “optimal” group, 7/26 patients (27%) experienced a downshift of the C score from baseline to 12 months of which 5 patients (71%) maintained “adequate” function, 1 (9.5%) worsened from “optimal” to “adequate,” and 1 (9.5%) maintained a “poor” function from 6 to 12 months.

Among the “adequate” group, 5/14 patients (35,7%) experienced an upshift to “optimal” and 1/14 patient (7%) a downshift to “poor” from baseline to 6 months for the C-score. Equally, 5/14 patients (35,7%) experienced an upshift to “optimal” and 1/14 patients (7%) a downshift to “poor” from baseline to 12 months for the C score. Of these 6 patients, 3 (50%) maintained an “optimal” function and 2 (33%) improved from “adequate” to “optimal” function from 6 to 12 months, whereas 1 patient (17%) downshifted from “adequate” to “poor” function from 6 to 12 months.

Among the “poor” group, 3/7 patients (43%) upshifted from “poor” to “optimal” function and 3/7 patients (43%) from “poor” to “adequate” function for C-score from baseline to 6 months. Indeed, 2/7 patients (28.5%) upshifted to “optimal” and 4/7 (57%) upshifted to “adequate” function for C-score from baseline to 12 months, respectively. Of these, 1 (14%) maintained an “optimal” function, 1 (14%) upshifted from “adequate” to “optimal,” 3 (43%) maintained an “adequate” function, and 1 (14%) downshifted from “optimal” to “adequate” from 6 to 12 months.

### Association Between ∆MDADI Score and Base of Tongue Cancer

No significant association for any covariate was found for ∆MDADI-C both in the “optimal” and “adequate/poor” groups from baseline to 6 months.

Indeed, a significant association was found in the “optimal” group for ∆MDADI-C score from baseline to 12 months for patients affected by base of tongue cancer (*p* = 0.031). Later, in the “optimal” group the presence of base of tongue cancer showed a statistically significant association with ∆MDADI of all subscale scores, except for F-item, from baseline to 12 months, as shown in Table 4.

**Table 4:** Association between base of tongue cancer and MDADI score variations from baseline to 12 months after treatment

	Base of tongue	Baseline MDADI ≥ 80			Baseline MDADI < 80			All patients	
		Mean	ds	*p*-value	Mean	ds	*p*-value	Mean (sd)	*p*-value
∆MDADI-E	No	− 9.28	13.82	0.053	12.92	16.26	0.731	0.03 (18.38)	0.528
	Yes	− 1.44	6.21		9.86	22.89		3.51 (16.24)	
∆MDADI-P	No	− 13.36	19.60	0.026	10.58	24.54	0.805	− 3.32 (24.55)	0.128
	Yes	− 0.71	6.88		13.06	19.87		7.03 (16.71)	
∆MDADI-F	No	− 8.36	12.82	0.142	17.78	15.38	0.927	− 0.77 (17.99)	0.385
	Yes	− 1.82	9.18		16.80	24.07		4,01 (17.03)	
∆MDADI-G	No	− 13.33	24.76	0.041	28.01	21.49	0.919	0,01 (30.55)	0.153
	Yes	4.01	8.43		26.67	30.11		12.51 (21.76)	
**∆**MDADI-C	No	− 10.83	14.85	0.031	11.35	19.041	0.930	− 1.53 (19.84)	0.257
	Yes	− 2.00	4.50		12.13	20.44		5.06 (16.05)	

∆ variation; *MDADI* M.D. Anderson Dysphagia Inventory; *E* emotional; *P* physical; *F* functional; *G* global; *C* composite; *ds* standard deviation

## Discussion

In this paper, we report the primary results of a multicenter still ongoing prospective trial endorsed by the Head and Neck Study Group (HNSG) of the Italian Association of Radiotherapy and Oncology (AIRO), aimed at investigating patient-reported deglutition outcomes after a SWOARs-sparing-based ChemoRT. Specifically, we report data regarding a primary endpoint for which the pre-planned number of patients has been achieved.

Overall, our study seems to show successful deglutition outcomes reporting a mean MDADI-C score of 79 and 81 at 6 and 12 months after treatment, regardless of the deglutition-related QoL at baseline. Hence, most patients seem to maintain an acceptable deglutition-related QoL suffering at worst from moderate disabilities (considered “adequate”), although severe ones (“poor”) were reported in a low but non-negligible percentage of patients. In fact, an MDADI-C score less than 60 (“poor”) was reported in 11% and 6.4% of patients at 6 and 12 months, respectively. This probably means that, after recovering from acute RT-related side effects, only a few patients perceive severe deglutition disorders. Thus, at first glance, our results on the overall patient population suggest an optimal preservation of deglutition-related QoL, reporting similar MDADI-C values between baseline and after treatment scores.

Actually, at a post hoc analysis a completely opposite trend was observed for all MDADI scores for patients with normal ability (“optimal”) compared with those with a certain degree of disability (“adequate” and “poor”) at baseline. Interestingly, “optimal” patients underwent a statistically significant worsening of all MDADI scores at 6 months followed by a slight non-statistically significant improvement at 12 months, according to most of the literature data on this issue [23–25]. On the contrary, “adequate/poor” patients experienced a statistically significant improvement of their function at 6 months followed by a subsequent stabilization at 12 months. Therefore, our findings seem to reveal a predominantly beneficial impact of ChemoRT on “cancer-related dysphagia.” This is probably due to the downsizing of the primary tumor mass that apparently overcomes its sequelae on the deglutition structures (“treatment-related dysphagia”). To our knowledge, this is the first study specifically reporting a beneficial impact of ChemoRT in patients with a certain degree of pre-treatment deglutition disorders and, in our opinion, the IMRT plan optimization to reduce dose to the SWOARs could play a decisive role in balancing the subtle equilibrium of these two opposite RT-induced adverse consequences.

Notwithstanding the fascinating clinical implication of our findings, the lack of robustness in the categorization of MDADI scores for the 3 different groups (“optimal,” “adequate,” “poor”) represents the main weakness of our study. In fact, despite this classification has already been reported in the previous major experience by Goepfert et al. [13] in order to facilitate the interpretation of clinical results, a real validation of the anchor points for this classification is yet to be done by the current literature. Anyway, despite the above-mentioned limitation, we honestly think that our findings might have interesting implications in clinical practice and are worth to be considered by clinicians. Moreover, a further major concern of our study was a proper interpretation of the longitudinal ∆MDADI scores in the two groups of patients to avoid misleading reports between statistical significance and clinical meaningfulness variations at different time points. In the study by Hutcheson et al. [22], a 10-point difference was defined as MCID to consider when using the MDADI as an outcome measure in clinical studies. Despite this, as declared by the authors themselves, the methodology used was designed to identify clinically relevant differences “between groups” rather than “within subjects.” As such, a general consensus on MCID for longitudinal ∆MDADI scores is yet to be defined in the scientific community. Thus, we decided to consider a “cut-off” value of 10-point difference as it is the only reference in the literature so far. In this regard, in the “adequate/poor” group, an overall increase of the MDADI-C score greater than 10 points (64.3 vs 76) was reported from before to after treatment suggesting a clinical meaningfulness other than a statistically significant improvement of deglutition-related QoL after RT. Besides, only 14% of patients maintained a poor C-score from baseline to both 6 and 12 months, whereas as much as 43% and 29% experienced an upshift to optimal function at 6 and 12 months, respectively. Therefore, a clinically relevant beneficial impact of ChemoRT on “cancer-related dysphagia” seems to emerge from our study. On the contrary, in the “optimal” group, an overall decrease of the MDADI-C score less than 10 points (93.6 vs 85.5) was reported from before to after treatment suggesting a statistically but not clinically meaningful worsening of the deglutition-related QoL. Besides, as much as 65% and 83% of patients maintained an optimal function for C-score at 6 and 12 months, whereas only 11% and 4% experienced a downshift from “optimal” to “poor” function for C score at 6 and 12 months, respectively. However, a clinical validation of the MCID cut-off value for longitudinal ∆MDADI scores (pre- vs post-treatment) is warranted for a proper interpretation of studies using the MDADI-C score as a primary clinical endpoint. However, we do recommend caution in the interpretation of our results that are limited by the use of a PRO metric, such as MDADI questionnaire, to measure the impact of a function-preserving radiation technique, such as SWOARs-sparing IMRT. In fact, at the moment our primary findings must be considered only as an “indirect” measurement of deglutition outcome that would need to be complemented by objective instrumental measures by means of Fiberoptic Evaluation of Swallowing (FEES) and/or Videofluoroscopy (VFS), specifically aimed to assess the deglutition function.

To date, most important experiences on this issue [13, 25, 26] are concordant in reporting a significant worsening of MDADI-C score early after treatment (mostly at 3 months) followed by a slow improvement at 6 and 12 months without returning to the baseline values, indicating a gradual partial recovery of deglutition-related QoL. The comparison of these data with ours might be troubled due to the opposite trend of MDADI-C scores between the two groups (“optimal” vs “adequate/poor”) that cancel the impact of treatment showing no significant differences in MDADI scores from before to after treatment. Indeed, we can strongly claim that in our study, the trend reported in the most experiences has been observed in the “optimal” MDADI group. Noteworthy, a recent experience by Grant et al. [27] using the more advanced intensity-modulated proton therapy (IMPT) technique, that significantly reduces dose to surrounding normal tissue compared with photon-based radiation, showed a rapid recovery of deglutition-related QoL at 10 weeks after treatment followed by a further steady improvement through 2 years achieving similar values of MDADI-C and subscale scores to the baseline.

Last but not least, patients affected with base of tongue cancer having a baseline “optimal” function seem to be most likely to suffer from RID. We observed a statistically significant association between the presence of this tumor subsite and variations of all but one (F-score) MDADI scale and subscale scores from baseline to 12 months after RT. It is noteworthy that, in a recent mono-institutional experience [28], the base of tongue has been identified as one of the most crucial SWOARs whose damage has been ranked of highest priority in the occurrence of post-deglutition inhalation. In this regard, a possible explanation of our findings might be the reduction of the posterior propulsive driving force of the base of tongue, due to the pre-treatment tumor infiltration of its musculature as well as to post-treatment fibrotic radiation damage, causing patients to perceive deglutition disorders. According to the most current literature [29–33], to address these patients to a continuous and early rehabilitation before, during, and early after treatment, together with the effort to recover as much as possible total oral nutrition (hopefully with semisolid or solid consistencies) might help reduce this self-reported deglutition disorders.

## Conclusion

Despite the lack of a MCID-validated cut-off for longitudinal ∆MDADI scores, our results seem to suggest a possible doubly clinical benefit of SWOARs-sparing IMRT both on the improvement of “Cancer-Related Dysphagia” as well as on the prevention of “Treatment-Related Dysphagia.” Of course, as PROs metrics such as MDADI questionnaire have shown to underestimate deglutition disorders compared to instrumental assessments [34], it will be interesting to correlate these findings with more objective measures, such as Fiberoptic Endoscopic Evaluation of Swallowing and Videofluoroscopy, that is within the additional endpoints of our study.

## Supplementary Information

Below is the link to the electronic supplementary material.

Supplementary file1 (PDF 51 KB)

## Data Availability

Research data are stored in our institutional repository and will be shared upon request to the corresponding author.

## References

[1] Siegel RL, Miller KD, Jemal A (2020). Cancer statistics. Am Cancer Soc.

[2] Pignon JP, Bourhis J, Domenge C, Designè L (2000). Chemotherapy added to locoregional treatment for head and neck squamous-cell carcinoma: three meta-analyses of updated individual data. MACH-NC Collaborative Group. Meta-analysis of chemotherapy on head and neck cancer. Lancet Lond Engl.

[3] Wilson JA, Carding PN, Patterson JM (2011). Dysphagia after non-surgical head and neck cancer treatment: patients’ perspectives. Head Neck Surg.

[4] Lovell SJ, Wong HB, Loh KS, Raymond YSN, Wilson JA (2005). Impact of dysphagia on quality-of-life in nasopharyngeal carcinoma. Head Neck.

[5] Nguyen NP, Frank C, Moltz CC, Vos P, Smith HJ, Karlsson U, Dutta S, Mydyett A, Barloon J, Sallah S (2005). Impact of dysphagia on quality of life after treatment of head and neck cancer. Int J Rad Oncol Biol Phys.

[6] Gluck I, Feng FY, Lyden T, Haxer M, Worden F, Chepeha DB, Eisbruch A (2010). Evaluating and reporting dysphagia in trials of chemoirradiation for head-and neck cancer. Int J Radiat Oncol Biol Phys.

[7] Raber-Durlacher JA, Brennan MT, Verdonck-de Leeuw IM, Gibson RK, Eilers JG, Sewnaik A, Bensadoun RJ, Fliedner MC, Silverman S, Spijkervet FKL (2012). Swallowing dysfunction in cancer patients. Support Cancer Care.

[8] Rosenthal DI, Lewin JS, Eisbruch A (2006). Prevention and treatment of dysphagia and aspiration after chemoradiation for head and neck cancer. J Clin Oncol.

[9] Chen AY, Frankowsky R, Bishop-Leone J, Hebert T, Leyk S, Lewin J, Goepfert H (2001). The development and validation of a dysphagia specific quality of life questionnaire for patients with head and neck cancer. Arch Otolaryngol Head Neck Surg.

[10] Patterson JM, McColl E, Carding PN, Hildreth AJ, Kelly C, Wilson JA (2014). Swallowing in the first year after chemoradiotherapy for head and neck cancer: clinician-and patient-reported outcomes. Head Neck.

[11] Cartmill B, Cornwell P, Ward E, Davidson W, Porceddu S (2012). A prospective investigation of swallowing, nutrition, and patient-rated functional impact following altered fractionation radiotherapy with concomitant boost for oropharyngeal cancer. Dysphagia.

[12] Lazarus CL, Husaini H, Hu K, Culliney B, Li Z, Urken M, Kacobson A, Persky M, Tran T, Concer C, Palacios C, Metcalfe-Klaw R, Kumar M, Bennet B, Harrison L (2014). Functional outcomes and quality of life after chemoradiotherapy: baseline and 3 and 6 months post-treatment. Dysphagia.

[13] Goepfert RP, Lewin JS, Barrow MP, Fuller CD, Lai SY, Song J, Hobbs BH, Brandon Gunn G, Beadle BM, Rosenthal DI, Garden AS, Merill SK, Papadimitrakopoulou VA, Schwartz DL, Hutcheson KA (2016). Predicting two-year longitudinal MD Anderson Dysphagia Inventory outcomes after intensity modulated radiotherapy for locoregionally advanced oropharyngeal carcinoma. Laryngoscope.

[14] Schwartz DL, Hutcheson K, Barringer D, Tucker SL, Kies M, Holsinger FC, Ang KK, Morrison HM, Rosenthal DI, Garden AS, Dong L, Lewin JS (2010). Candidate dosimetric predictors of long-term swallowing dysfunction following oropharyngeal IMRT. Int J Radiat Oncol Biol Phys.

[15] Orlandi E, Miceli R, Infante G, Mirabile A, Alterio D, Cossu Rocca M, Denaro N, Vigna-Taglianti R, Merlotti A, Schindler A, Pizzorni N, Fallai C, Licitra L, Bossi P (2019). Predictors of patient-reported dysphagia following IMRT plus chemotherapy in oropharyngeal cancer. Dysphagia.

[16] Ursino S, D’Angelo E, Mazzola R, Merlotti A, Morganti R, Cristauda A, Paiar F, Musio D, Alterio D, Bacigalupo A, Russi EG, Lohr F (2017). A comparison of swallowing dysfunction after three-dimensional conformal and intensity-modulated radiotherapy: a systematic review by the Italian head and neck radiotherapy study group. Strahlenther Onkol.

[17] Eisbruch A, Hyungjin MK, Feng FY, Lyden TH, Haxer MJ, Feng M, Worden FP, Bradford CR, Prince ME, Moyer JS, Wolf GT, Chepeha DB, Haken RKT (2011). Chemo-IMRT of oropharymgeal cancer aiming to reduce dysphagia: swallowing organs late complication probabilities and dosimetric correlates. Int J Radiat Oncol Biol Phys.

[18] Christianen M, Van der Schaaf A, Van der Laan H, Verdonck-de Leeuw I, Doornaert P, Chouvalova O, Steenbakkers RJHM, Leemas CR, Osting SF, Van Der Laan BFAM, Roodenburg JLN, Slotman BJ, Bijl H, Langendijk JA (2016). Swallowing sparing intensity modulated radiotherapy (SW-IMRT) in head and neck cancer: clinical validation according to the model-based approach. Radiother Oncol.

[19] Merlotti A, Alterio D, Vigna Taglianti R, Muraglia A, Lastrucci L, Manzo R, Gambaro G, Caspiani O, Miccichè F, Deodato F, Pergolizzi S, Franco P, Corvò R, Russi EG, Sanguineti G (2014). Technical guidelines for head and neck cancer on behalf of the Italian association of radiation oncology-head and neck working group. Radiat Oncol.

[20] Christianen ME, Langendijk JA, Westerlaan HE, VanDerWater TA, Bijl HP (2011). Delineation of organs at risk involved in swallowing for radiotherapy treatment planning. Radiother Oncol.

[21] Schindler A, Borghi E, Tiddia C, Ginocchio D, Felisati G, Ottaviani F (2008). Adaptation and validation of the Italian MD Anderson dysphagia inventory (MDADI). Rev Laryngol Otol Rhinol.

[22] Hutcheson KA, Portwood M, Lisec A, Barringer DA, Gries K, Lewin JS (2016). What is a clinically relevant difference in MDADI scores between groups of head and neck cancer patients?. Laryngoscope.

[23] Lazarus CL, Husaini H, Hu K, Culliney B, Li Z, Urken M, Jacobson A, Persky M, Tran T, Concert C, Palacios D, Metcalfe-Klaw R, Kumar M, Bennet B, Harrison L (2014). Functional outcomes and quality of life after chemoradiotherapy: baseline and 3 and 6 months post-treatment. Dysphagia.

[24] Schwartz DL, Hutcheson K, Barringer D, Tucker SL, Kies M, Holsinger FC, Ang KK, Morrison WH, Rosenthal DI, Garden AS, Dong L, Lewin JS (2010). Candidate dosimetric predictors of longterm swallowing dysfunction following oropharyngeal IMRT. Int J Radiat Oncol Biol Phys.

[25] Jovanovic N, Dreyer C, Hawkins S, Thouless K, Palma D, Doyle PC, Theurer JA (2021). The natural history of weight and swallowing outcomes in oropharyngeal cancer patients following radiation or concurrent chemoradiation therapy. Support Care Cancer.

[26] Roe JWG, Drinnan MJ, Carding PN, Harrington KJ, Nutting CM (2014). Patient-reported outcomes following parotid-sparing intensity-modulated radiotherapy for head and neck cancer. How important is dysphagia?. Oral Oncol.

[27] Grant SR, Hutcheson KA, Ye R, Garden AS, Morrison WH, Rosenthal DI, Gunn GB, Fuller CD, Phan J, Reddy JP, Moreno AC, Lewin JS, Sturgis EM, Ferrarotto R, Frank SJ (2020). Prospective longitudinal patient-reported outcomes of swallowing following intensity modulated proton therapy for oropharyngeal cancer. Radiother Oncol.

[28] Ursino S, Giuliano A, DiMartino F, Cocuzza P, Molinari A, Stefanelli A, Giusti P, Aringhieri G, Morganti R, Neri E, Traino C, Paiar F (2021). Incorporating dose–volume histogram parameters of swallowing organs at risk in a videofluoroscopy-based predictive model of radiation-induced dysphagia after head and neck cancer intensity-modulated radiation therapy. Strahlenther Onkol.

[29] King SN, Dunlap NE, Tennant PA, Pitts T (2016). Pathophysiology of radiation-induced dysphagia in head and neck cancer. Dysphagia.

[30] Carnaby-Mann G, Crary MA, Schmalfuss I, Amdur R (2012). “Pharyngocise”: randomized controlled trial of preventative exercises to maintain muscle structure and swallowing function during head-and neck chemoradiotherapy. Int J Radiat Oncol Biol Phys.

[31] Hutcheson KA, Bhayani MK, Beadle BM, Gold KA, Shinn EH, Lai SY, Lewin J (2013). Eat and exercise during radiotherapy or chemoradiotherapy for pharyngeal cancers: use it or lose it. JAMA Otolaryngol Head Neck Surg.

[32] De Felice F, DeVincentiis M, Luzzi V, Magliulo G, Tombolini M, Ruoppolo G, Polimeni A (2018). Late radiation-associated dysphagia in head and neck cancer patients: evidence, research and management. Oral Oncol.

[33] Schindler A, Denaro N, Russi EG, Pizzorni N, Bossi P, Merlotti A, Spadola Bissetti M, Numico G, Gava A, Orlandi E, Caspiani O, Buglione M, Alterio D, Bacigalupo A, De Sanctis V, Pavanato G, Ripamonti C, Merlano M, Licitra L, Sanguineti G, Langendijk JA, Murphy B (2015). Dysphagia in head and neck cancer patients treated with radiotherapy and systemic therapies: literature review and consensus. Crit Rev Oncol Hematol.

[34] Jensen K, Lambertsen K, Torkov P, Dahl M, Jensen AB, Grau C (2007). Patient assessed symptoms are poor predictors of objective findings. Results from a cross sectional study in patients treated with radiotherapy for pharyngeal cancer. Acta Oncol.

