# Supplementary material for: Patient-Reported Outcomes After Swallowing (SWOARs)-Sparing IMRT in Head and Neck Cancers: Primary Results from a Prospective Study Endorsed by the Head and Neck Study Group (HNSG) of the Italian Association of Radiotherapy and Clinical Oncology (AIRO)
Source: Dysphagia. 2022 May 19;38(1):159–70. doi: 10.1007/s00455-022-10434-4 (PMC9118191; doi:10.1007/s00455-022-10434-4)
Supplement: Supplementary file 1 — Supplementary file1 (PDF 51 KB) [file 455_2022_10434_MOESM1_ESM.pdf]

RADIOTHERAPY PLANNING CRITERIA

Target volume delineation and dose fractionation

Two mandatory different target volumes are defined as an high-risk and a low-risk planning target volume (HR-PTV and LR-PTV) whereas the intermediate-risk (IR-PTV) is considered optional.

The prescription dose to the different volumes has to be delivered by using a simultaneous integrated boost (SIB)-IMRT technique for a total of 30 fractions as follows:

- HR-PTV= 66Gy (2.2Gy/fraction)
- IR-PTV=60Gy (2.0Gy/fraction)
- LR-PTV (1.8Gy/fraction)

Criteria for definition of high-risk target volumes for nasopharynx and oropharynx are reported in Table 1.

Table 1 Contouring criteria of high-risk target volumes

**Target Volumes**	**Definition and description**
GTV_{66}	Primary: "Gross disease" on physical examination and imaging (CT,MR and fibroscopy)<br><br>Neck nodes: all nodes ≥ 1cm or those with necrotic center
CTV_{66}	GTV_{66} + 3 mm circumferential margin or less in case of overlapping with primary organs at risk or in presence of anatomic boundaries
PTV_{66}	CTV_{66} + 3mm circumferential margin or less in case of overlapping with primary organs at risk or in presence of anatomic boundaries

Criteria for definition of low-risk target volumes for nasopharynx and oropharynx are reported in Table 2 and 3.

Table 2 Contouring criteria of low-risk target volumes for nasopharynx

**Target Volumes**	**Definition and description**
CTV_{54}	CTV_{66} + 5 mm margin and region at risk for microscopic which include:<br>▪ Entire nasopharynx<br>▪ Anterior 1/3 of the clivus (entire clivus if involved)<br>▪ Skull base<br>▪ Pterigoyd fossa<br>▪ Parapharyngeal space<br>▪ Inferior sphenoid sinus (entire sphenoid sinus in T3-T4 disease)<br>▪ Posterior 1/4 of the nasal cavità/maxillary sinuses<br>▪ Inferior soft palate<br>▪ Retropharyngeal lymph nodes<br>▪ Retrostyloid space<br>▪ Bilateral nodal levels Ib through V [a]
PTV_{54}	CTV $_{54}$ + 3-5 mm margin or less in case of overlapping with primary organs at risk

[a] Level Ib can be omitted in case of node-negative disease

Table 3 Contouring criteria of low-risk target volumes for oropharynx

**Target Volumes**	**Definition e Description**
CTV $_{54}$	Primary: CTV_{66} + 5 mm margin<br><br>Nodes:<br>▪ Levels II-IV<br>▪ Lateral retropharyngeal nodes up to skull base/jugular foramen (node-positive neck) or up to C1 (node-negative neck)<br>▪ High level II/retrostyloid space (node-positive neck)
PTV $_{54}$	CTV $_{54}$ + 3-5 mm or less in case of overlapping with primary organs at risk

An optional intermediate-risk CTV (CTV_{60}) can be defined by the pysichian corrisponding to area at high risk for microscopic tumor involvement (>10-20%) such as regions surrounding primary tumor or suspected lymph-nodes.

A 3mm isotropic expansion is added to define $PTV_{60.}$

Treatment plan evaluation

Table 4 Treatment plan evaluation criteria

**Target Volume**	**Definition and Description**
PTV Coverage	D95 ≥ 95% prescription dose
Dose Homogeneity	D05 $PTV_{66} \leq$ 110%<br><br>D05 $PTV_{54} \leq$ prescription dose to HR-PTV*

* This constraint is referred to subtraction of volumes (PTV_{54}-PTV_{66})

Constraints of Primary Organs at Risk (P-OARs)

Dosimetric constraints to primary organs at risk are reported in Table 5.

For treatment planning, brainstem, spinal cord and other nerve structures (optic nerve and chiasm) must be defined as PRV (Planning Risk Volume) by adding a 3 mm isotropic expansion.

Table 5 Constraints of primary organs at risk (P-OARs)

**Primary Organs at Risk**	**Volume**	**Constraints**
Brainstem	0.1cc	D_{max} 54Gy
Temporal lobes	1cc	D_{max} 60Gy
Spinal cord	0.1cc	$D_{max} \leq$ 44-45Gy
Chiasm	0.1cc	D_{max} 54Gy
Optic nerves	0.1cc	D_{max} 54Gy
Mandibule	1cc	Dmax 70-73.5Gy
Brachial Plexus	0.1cc	Dmax 60Gy

Constraints of Secondary Organs at risk (S-OARs)

Swallowing-related structures (SWOARs), oral cavity (OC), parotid glands (PGs) and submandibular glands (SMGs) are defined as S-OARs. Definition of PRV is not required for these structures.

Dosimetric constraints for Secondary Organs at Risk are reported in Table 6.

Table 6 Dosimetric constraints of SWOARs

SWOARs	Constraints
SPCM, MPCM e IPCM	Dm<63Gy (TDs50); Dm<56Gy (TDs25)
BOT	Dm ≤ 55Gy
SL	Dm<56Gy (TDs50); Dm<39Gy (TDs25)
GL	Dm≤45Gy; V50<21%
CPM	Dm<40Gy
EC	Dm<40Gy

Abbreviations: SPCM: Superior pharyngeal constrictor muscle; MPCM: Middle pharyngeal constrictor muscle; IPCM: Inferior pharyngeal constrictor muscle; BOT: Base of tongue; SL: Supraglottic larynx; GL: Glottic larynx; CPM: Cricopharyngeal muscle; EC: Cervical esophagus; Dm= mean dose

Regarding the others S-OARs, the following dosimetric constraints are recommended, if possible:

Unilateral parotid gland: V30<50%; Dm≤ 26Gy

Bilateral parotid gland: Dm<39Gy

Anterior oral cavity: V30<65%; V35<35%

Submandibular gland: Dm<35Gy
